# Mixed infections with distinct cytomegalovirus glycoprotein B genotypes in Polish pregnant women, fetuses, and newborns

**DOI:** 10.1007/s10096-014-2266-9

**Published:** 2014-10-28

**Authors:** M. Rycel, W. Wujcicka, B. Zawilińska, E. Paradowska, P. Suski, Z. Gaj, J. Wilczyński, Z. Leśnikowski, D. Nowakowska

**Affiliations:** 1 Department of Gynecology and Gynecologic Oncology, Polish Mother’s Memorial Hospital Research Institute, 281/289 Rzgowska Street, Lodz, 93-338 Poland; 2 Department of Fetal-Maternal Medicine and Gynecology, Polish Mother’s Memorial Hospital Research Institute, 281/289 Rzgowska Street, Lodz, 93-338 Poland; 3 Department of Fetal-Maternal Medicine and Gynecology, Chair of Obstetrics, Gynecology and Gynecologic Oncology, Medical University of Lodz, Lodz, Poland; 4 Department of Virology, Jagiellonian University Medical College, Cracow, Poland; 5 Laboratory of Molecular Virology and Biological Chemistry, Institute of Medical Biology, Polish Academy of Sciences, Lodz, Poland

## Abstract

The purpose of this investigation was to describe a distribution of cytomegalovirus (CMV) single and multiple genotypes among infected pregnant women, their fetuses, and newborns coming from Central Poland, as well as congenital cytomegaly outcome. The study involved 278 CMV-seropositive pregnant women, of whom 192 were tested for viral DNAemia. Human cytomegalovirus (HCMV) genotyping was performed for 18 of 34 pregnant women carrying the viral DNA and for 12 of their 15 offspring with confirmed HCMV infections. Anti-HCMV antibodies levels were assessed by chemiluminescence immunoassay (CLIA) and enzyme-linked fluorescence assay (ELFA) tests. Viral DNA loads and genotypes were determined by real-time polymerase chain reaction (PCR) assays for the *UL55* gene. In the pregnant women, we identified HCMV gB1, gB2, gB3, and gB4 genotypes. Single gB2, gB3, or gB4 genotypes were observed in 14 (77.8 %) women, while multiple gB1–gB2 or gB2–gB3 genotypes were observed in four (22.2 %). Maternal HCMV genotypes determined the genotypes identified in their fetuses and newborns (*p* ≤ 0.050). Half of them were infected with single HCMV gB1, gB2, or gB3 genotypes and the other half with multiple gB1–gB2 or gB2–gB3 genotypes. Single and multiple genotypes were observed in both asymptomatic and symptomatic congenital cytomegaly, although no gB3 genotype was identified among asymptomatic cases. In Central Poland, infections with single and multiple HCMV strains occur in pregnant women, as well as in their fetuses and neonates, with both asymptomatic and symptomatic infections. HCMV infections identified in mothers seem to be associated with the viral genotypes in their children.

## Introduction

Human cytomegalovirus (HCMV) is the most common cause of intrauterine infections worldwide [1–5]. Nucleotide variability was determined for approximately 20 open reading frames (ORFs) of HCMV that encode, e.g., viral envelope glycoproteins B (gB, *UL55*), H (gH, *UL75*), and N (gN, *UL73*), as well as chemokines and chemokine receptors, like a tumor necrosis factor (TNF)-α receptor (pUL144, *UL144*) [6–9]. Although sequence variability in the large genome generates extensive viral strain diversity, the clinical significance of HCMV genotypes remains rather unknown.

Glycoprotein B is the major HCMV envelope protein that has been determined as an important factor for viral in vivo and in vitro replication, as well as for host cell entry, cell-to-cell virus transmission, and fusion of infected cells [4, 10–12]. The gB polypeptide, composed of 906 amino acids, is translated into a 160-kDa precursor molecule that is cleaved at position 460 by cellular endoprotease to produce the N- and C- terminal products [11, 13, 14]. The cleavage site and the region between 448 and 481 codons were described as the area of the highest genetic variability [11, 14]. Considering this highly polymorphic site, four main HCMV genotypes, gB1, gB2, gB3, and gB4, and three rare non-prototypic variants, gB5, gB6, and gB7, were defined [11, 14]. A recent study, performed in newborns from Central Poland showed *UL55* gB2 as the most prevalent genotype of HCMV (96 %) [4]. In contrast, an earlier study performed in infants and newborns from Southern Poland demonstrated gB1 (63.5 %) as the most common genotype [14].

Some researchers have also reported relationships between HCMV genotypes and the development and outcome of cytomegaly, observed in human immunodeficiency virus (HIV)-infected patients, hematopoietic transplant recipients, as well as in congenitally infected fetuses and newborns [15–17]. However, some reports indicated no association between gB genotypes and cytomegaly severity in either fetuses or newborns [18–21].

In this study, we determined the gB genotypes of HCMV in pregnant Polish women, infected during pregnancy, as well as in their fetuses and newborns with confirmed congenital infections. Moreover, we investigated if any coinfections with different HCMV genotypes might have occurred in the analyzed groups coming from Central Poland. The primary goal of our study was to search for viral genotypes transmitted from mother to fetus through the placenta. The secondary goal included an attempt to describe the relationship between the occurrence of mixed infections and cytomegaly outcomes.

## Materials and methods

**Fig. 1:**
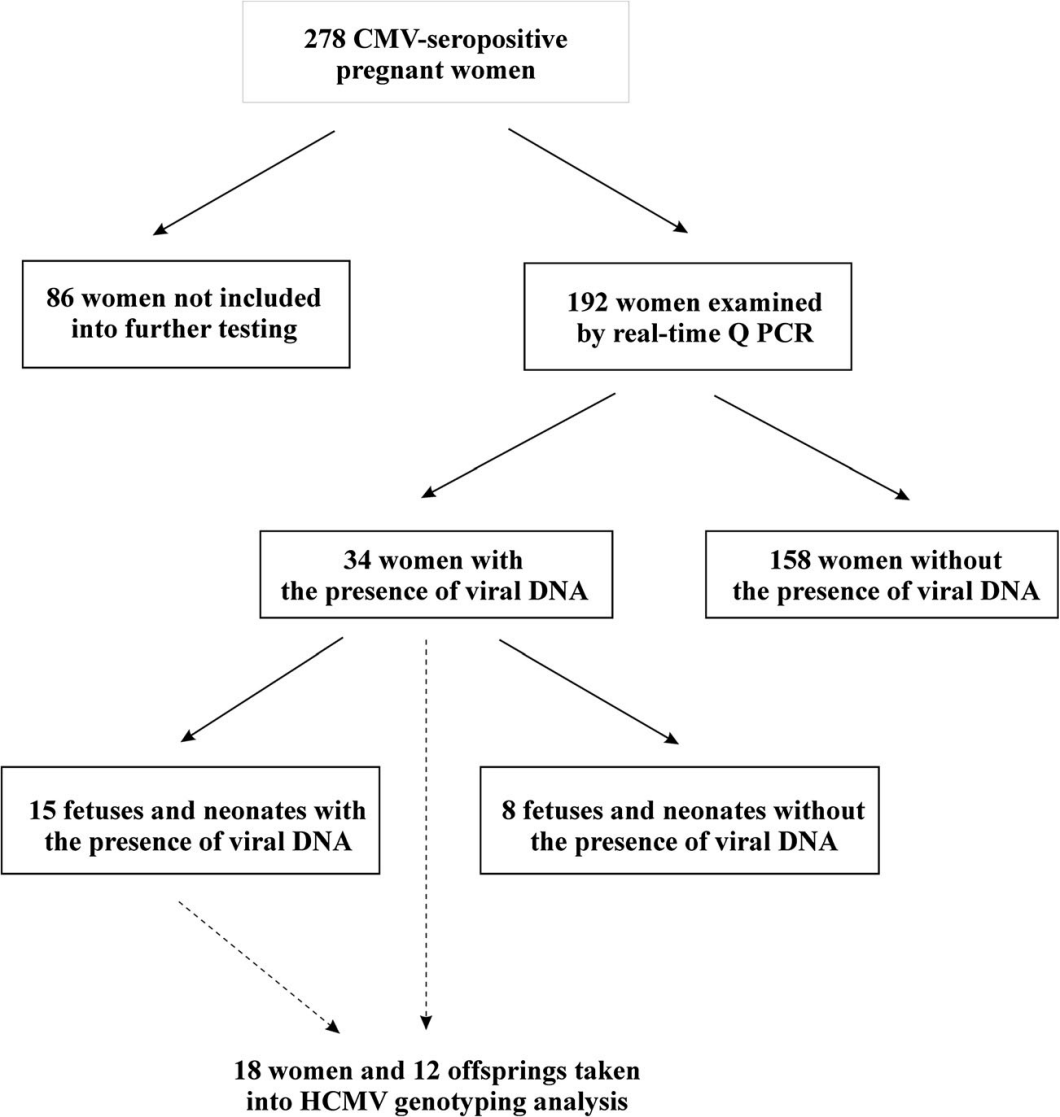
Distribution of pregnant women, their fetuses, and newborns included in the serological testing for human cytomegalovirus (HCMV) infections and genotyping of the virus

The study group consisted of 278 CMV-seropositive pregnant women admitted to the Department of Fetal-Maternal Medicine and Gynecology at the Polish Mother’s Memorial Hospital Research Institute in Lodz between September 2009 and July 2013 (see Figure 1). Of the total study population, 192 women were tested for HCMV DNAemia. The presence of viral DNA was determined for 34 examined patients. Among the fetuses and newborns of mothers carrying HCMV DNA, the viral load was studied for 23 cases, of which 15 were viral DNA-positive. The isolated HCMV DNA solutions enabled viral genotyping in 18 pregnant women, as well as in their 12 offspring with confirmed HCMV infections. For two out of all fetal cases, we acquired further data on the viral genotype status, determined also at their postnatal stage. Six fetuses and one newborn presented with symptomatic congenital cytomegaly. Of the symptomatic fetuses, three were fatal cases. The asymptomatic cytomegaly in fetuses and newborns was determined in case of the lack of any ultrasound symptoms related to the disease, while the symptomatic cytomegaly was determined in case of the occurrence of at least one cytomegaly-associated ultrasound marker. The ultrasound markers related to cytomegaly included ventriculomegaly, hydrocephaly, and fetal hydrops, as well as intrauterine growth restriction (IUGR), ascites, pericardial effusion, cardiomegaly, and hyperechogenic foci in various organs.

All the women included the study had serological features of a possible recent infection with HCMV, identified during pregnancy. The patients with determined specific IgM as well as low IgG avidity were suspected as primary infected with HCMV. The kinetics of specific IgG were analyzed to determine the occurrence of the infection as well. The preliminary diagnosis was also based on disease-related clinical features, including flu-like symptoms in the pregnant women and newborns, as well as ultrasound markers observed in the fetuses and neonates. Active infection in pregnant women was confirmed by the presence of HCMV DNA in body fluids, including blood and urine. Regarding the fetuses, viral DNA was identified in amniotic fluids/cells, umbilical cord blood, or ascitic fluids, while in neonates it was identified in blood or urine specimens, as well as samples of the brains, kidneys, and livers in the fatal cases. Data about the genotypes and DNA loads of HCMV, determined in the body fluids of two fetuses and newborns, have been published in our recent paper [4].

### Serological tests

Blood samples from the pregnant women were taken by venipuncture during their visit to the institute between the 8th and 39th week of gestation and from infants within a day of birth. Serum fractions were obtained by centrifugation and stored at 4°C until analysis. Serological tests were performed at the Department of Clinical Microbiology of the Polish Mother’s Memorial Hospital.

Between the years 2009 and 2011, the screening for HCMV IgG and IgM antibodies and for IgG avidity was performed by the chemiluminescence immunoassay (CLIA), using anti-CMV IgG and IgM tests (DiaSorin/Biomedica, Italy). Samples were considered as IgG- or IgM-positive for antibody levels ≥0.6 IU/ml and ≥30 IU/ml, respectively. The IgG avidity with indexes <0.200 was interpreted as low, 0.200–0.300 as borderline, and ≥300 as high. From the year 2012, the CLIA method was replaced by the enzyme-linked fluorescence assay (ELFA). IgG antibody levels ≥6 IU/ml and IgM indexes ≥0.9 were considered positive. The pregnant women were suspected as HCMV-infected in case of IgG seroconversion during pregnancy, IgM seropositivity, and low IgG avidity index. In those patients, the presence of viral DNA was determined by a real-time quantitative polymerase chain reaction (Q PCR) assay for the viral *UL55* gene in blood, urine, and amniotic fluids.

### DNA isolation

HCMV DNA was extracted from maternal whole blood, plasma, peripheral blood mononuclear cells (PBMCs), and urine. For the studies of congenital infections, viral DNA was isolated from amniotic and ascitic fluids of the fetuses, as well as from plasma, whole blood, and urine of the newborns. In the fatal cases, autopsy was performed, followed by an analysis of DNA, purified from fragments of brains, kidneys, and livers, stored as paraffin-embedded tissues. Total DNA from all the clinical specimens was extracted with a QIAamp DNA Mini Kit (Qiagen, Hilden, Germany). The concentration and purity of extracted DNA were assessed by spectrophotometry, using a NanoDrop ND-1000 Spectrophotometer (NanoDrop Technologies, Inc., Wilmington, DE, USA).

### Quantification of HCMV DNA

The amount of HCMV DNA in the clinical samples was determined by the real-time Q PCR assay for the detection of *UL55* gene fragment, as described previously [22, 23]. The reactions were done in triplicate and the PCR conditions were as follows: initial activation for 10 min at 95 °C and 50 cycles of repeated denaturation at 95 °C for 15 s and annealing at 60 °C for 1 min. The standard curves, used in the quantitative analyses, were obtained from serial 10-fold dilutions from 10^5^ to 1 HCMV DNA copy. The amplification was performed by a 7900HT Fast Real-Time PCR System (Applied Biosystems, USA). HCMV DNA determination was performed for one to five different types of samples for each patient suspected to be infected with the virus during pregnancy, according to the availability of the clinical materials.

### HCMV glycoprotein B genotyping

Four *UL55* genotypes of HCMV were distinguished by the real-time PCR assay, using previously described primers and probes [24]. The PCR conditions were as follows: initial denaturation for 20 s at 95 °C and 40 cycles of repeated denaturation at 95 °C for 3 s and annealing at 60 °C for 30 s. The real-time PCR assays were performed using the 7500 Fast Real-Time PCR System (Applied Biosystems, USA). In all reactions, the serial dilutions of HCMV reference strains Towne (gB1; ATCC: VR-977) and AD-169 (gB2; ATCC: VR-538) were included to plot calibration curves. HCMV DNA extracted from one to five different types of clinical specimens for each of the patients was used for viral genotyping, depending on the presence of viral DNA determined by the real-time Q PCR assay.

### Statistical analysis

The distribution of particular gB genotypes of HCMV, observed in pregnant women, their fetuses, and newborns, was assessed by means of descriptive statistics. The relationships between HCMV genotypes, identified in pregnant women and genotypes, determined in fetuses and neonates, as well as those between the viral genotype and the fetal outcome of the disease, were estimated using cross-tabulation and Pearson’s Chi-squared or Fisher’s exact tests. All the results were defined as statistically significant at the significance level of *p* ≤ 0.050. The data were analyzed using the NCSS 97 software.

## Results

### HCMV *UL55* genotypes among pregnant women and their fetuses and neonates

In maternal body fluids, there were identified four main *UL55* genotypes of HCMV. The gB1, gB2, and gB3 genotypes were determined in whole blood, plasma, PBMCs, and urine specimens, while the gB4 genotype was found only in urine. Considering whole blood, the single gB1 genotype was observed in 10.0 % (1/10) of HCMV DNA-positive samples and gB2 in 80.0 % (8/10), while multiple gB2–gB3 genotypes were observed in 10.0 % (1/10). In plasma, the single gB2 genotype was observed in 50.0 % of women (2/4), while multiple gB1–gB2 or gB2–gB3 genotypes were observed in 25.0 % (1/4). In PBMCs, the single gB2 genotype was present in 60.0 % of patients (3/5) and gB3 in 20.0 % (1/5), while multiple gB1–gB2 genotypes were observed in 20.0 % (1/5). Of all HCMV-positive urine samples, 64.3 % (9/14) possessed the single gB2 genotype and 14.3 % (2/14) possessed the single gB4 or multiple gB1–gB2 genotypes, while 7.1 % (1/14) possessed multiple gB2–gB3 genotypes.

Taking into account the all HCMV-positive pregnant women, infections with single HCMV genotypes were observed in 77.8 % (14/18), while mixed infections were observed in 22.2 % (4/18) of the study population (see Table 1). Taking into account maternal viral genotypes, the highest incidence was determined for the gB2 genotype, identified in 61.1 % (11/18), and there were lower incidence rates for the gB4 and gB3 genotypes, observed in 11.1 % (2/18) and 5.55 % (1/18) of the pregnant women, respectively. Among the mixed infections, viral gB1–gB2 genotypes were noticed in 16.7 % (3/18), while the gB2–gB3 genotypes were found in 5.55 % (1/18) of the pregnant women. Considering the single and mixed infections together, the gB2 genotype was identified in 77.8 % (14/18) of women and gB1 in 16.7 % (3/18), while both gB3 and gB4 were observed in 11.1 % (2/18). Of seven pregnant women with assessed HCMV genotypes in their fetuses and newborns four had the gB2 genotype, two had gB1–gB2 genotypes, and one had gB2–gB3 genotypes (see Table 2). Four fetuses of the mothers infected with the gB2 genotype of HCMV revealed the same genotype. Similar results were observed in the triplets of one mother and in another fetus of a different mother, both mothers being infected with multiple HCMV gB1–gB2 genotypes. The fetus of the mother with multiple gB2–gB3 genotypes had only the gB2 genotype. However, the gB3 genotype was found in the maternal body fluids (plasma, whole blood, urine) of another pregnant woman after gB2 genotype identification in the amniotic fluid. HCMV genotypes identified in the pregnant women were estimated to have significantly determined the viral genotypes congenitally acquired by the fetuses (*p* ≤ 0.050; see Table 2).

**Table 1:** Distribution of human cytomegalovirus (HCMV) genotypes among the examined pregnant women, fetuses, and newborns

Study group	Total no. tested*	HCMV *UL55* genotype [no. tested (%)**]							
		Single				All single infections	Multiple		All multiple infections
		gB1	gB2	gB3	gB4		gB1–gB2	gB2–gB3	
Pregnant women	18	0 (0)	11 (61.1)	1 (5.55)	2 (11.1)	14 (77.8)	3 (16.7)	1 (5.55)	4 (22.2)
Fetuses	9	1 (11.1)	6 (66.7)	0 (0)	0 (0)	7 (77.8)	1 (11.1)	1 (11.1)	2 (22.2)
Newborns	5	0 (0)	1 (20)	0 (0)	0 (0)	1 (20)	4 (80)	0 (0)	4 (80)
Congenital infections	12	1 (8.3)	5 (41.7)	0 (0)	0 (0)	6 (50)	5 (41.7)	1 (8.3)	6 (50)
Symptomatic fetuses and newborns	6	1 (16.66)	3 (50)	0 (0)	0 (0)	4 (66.7)	1 (16.66)	1 (16.66)	2 (33.3)
Asymptomatic fetuses and newborns	6	0 (0)	2 (33.3)	0 (0)	0 (0)	2 (33.3)	4 (66.7)	0 (0)	4 (66.7)

*Number of patients tested

**Percentage of all patients included in each study group

**Table 2:** Relationship between HCMV genotypes identified in the pregnant women and their offspring

Maternal genotype	Fetal and neonatal genotype		Total
	gB2	gB1–gB2	
gB2	4	0	4
gB1–gB2	0	4	4
gB2–gB3	1	0	1
Total	5	4	9

χ^2^ = 9

*p* ≤ 0.050

### HCMV *UL55* genotypes in fetal and neonatal infections

In the infected fetuses and newborns, the gB1, gB2, and gB3 genotypes of HCMV were observed in various studied clinical specimens. Taking into account the amniotic fluids, the single gB2 genotype was determined in 75.0 % (6/8) of HCMV-positive specimens, while the multiple gB1–gB2 or gB2–gB3 genotypes were observed in 12.5 % (1/8). Of two HCMV-positive ascitic fluids, the first possessed the gB2 genotype and the second possessed the multiple gB1–gB2 genotypes. All three analyzed whole-blood and one urine samples carried the multiple gB1–gB2 genotypes. Of two plasma specimens, the first possessed the single gB2 genotype and the second possessed the multiple gB1–gB2 genotypes. Most fetuses and neonates had the same HCMV genotype identified in all of the analyzed samples. Comparison of the distribution of single versus multiple HCMV genotypes between distinct clinical specimens showed a marginally significant difference (χ^2^ = 15; *p* = 0.0592). The multiple viral genotypes were significantly more frequent in fetal and neonatal body fluids than in tissue fragments [100.0 % (8/8) vs. 0.0 % (0/8); *p* ≤ 0.050; Fisher’s exact test].

Of all congenital infections, half (6/12) were caused by single HCMV genotypes and the other half by multiple genotypes (see Table 1). A single gB1 genotype was determined in 11.1 % (1/9) of the fetuses but not in any of the newborns. The gB2 genotype was identified in 66.7 % (6/9) of the fetuses and in 20.0 % (1/5) of the newborns. Coinfections with the gB1–gB2 genotypes of HCMV were determined in 11.1 % (1/9) and 80.0 % (4/5) of the fetuses and newborns, respectively. Mixed infections with the gB2–gB3 genotypes were observed in 11.1 % of the fetuses. In contrast, this type of infection was identified in none of the newborns included in the study. Taking into account the single and mixed infections together, both gB1 and gB2 genotypes of HCMV were determined in 50.0 % (6/12) of the congenitally infected fetuses and newborns, while gB3 was observed in 8.3 % (1/12) of cases.

### Cytomegaly outcome in fetuses and newborns with distinct HCMV genotypes

Among all fetal and neonatal infections, 50.0 % (6/12) were asymptomatic and the other 50.0 % were symptomatic (see Table 1). The fetuses and newborns with asymptomatic cytomegaly had single gB1, gB2, or multiple gB1–gB2 genotypes. Two cases (33.0 %) were infected with a single HCMV gB2 genotype, while four (66.7 %) were infected with multiple gB1–gB2 genotypes. The fetuses and neonates with symptomatic disease had all three HCMV genotypes determined. Among them, a single gB2 genotype was identified in 50.0 % (3/6) of cases, while the gB1 genotype was identified in 16.66 % (1/6). Mixed infections with the gB1–gB2 or gB2–gB3 genotypes of HCMV were determined in 16.66 % of the fetuses and newborns for each type of infection.

## Discussion

Intrauterine HCMV infections and congenital cytomegaly are caused by four gB genotypes of HCMV [19, 25, 26]. In this reported study, we identified HCMV gB1, gB2, and gB3 genotypes in some Polish fetuses and newborns. Similarly, congenital infections among Costa Rican, Indian, and Chinese infants were caused by the same three viral genotypes [26–28]. Studies performed in HCMV-infected newborns and infants from Southern Poland revealed the occurrence of gB1, gB2, gB3, and gB4 viral genotypes in 43.7 %, 31.25 %, 25 %, and 12.5 % of children, respectively [14]. In our study, we observed slightly higher incidence rates of gB1 and gB2 genotypes (50.0 %) and lower rates for the gB3 genotype (8.3 %), while no gB4 genotype was found among either the fetuses or the newborns. Similar frequencies of HCMV genotypes were reported also for Costa Rican and Indian infants [20, 27]. Differences observed between our results and the results of the study of newborns and infants from Southern Poland may not be significant, since both study groups were relatively small. Therefore, research would be justified with a larger group of fetuses, newborns, and infants. In contrast, the recent study of newborns from Central Poland demonstrated gB2 to be the major genotype (96 %) of HCMV among infected neonates [4]. Since the samples used in both studies included offspring born in the same hospital during a similar period of time, we suggest different sensitivity of methods as well as variety of types of clinical materials used in the described research for the determination of HCMV genotypes as the main reasons for the discrepancies observed [4, 24]. In our study, most cases of the gB1 genotype of HCMV (83.3%, five of six cases) were involved in mixed infections with gB1–gB2 genotypes. Taking into account the ability of direct sequencing to select one strain only, it seems possible that, in such cases, the previously adapted method could also find only a single gB2 strain of HCMV [24]. However, comparison of the results obtained for two offspring analyzed in both our studies showed that different genotypes identified might come from the different types of clinical materials used as well. For two mentioned fetuses, the current study showed the presence of HCMV gB1–gB2 or gB2–gB3 multiple genotypes in urine specimens or amniotic/ascitic fluids, respectively. In turn, the previous study performed for neonatal blood samples collected on the day of birth reported that investigated offspring were infected with the viral gB2 or gB3 genotypes, respectively [4]. Therefore, the use of assays with the highest sensitivity and various clinical specimens seems to be necessary to determine the whole spectrum of HCMV genotypes related to congenital infections.

The incidence rates of gB1 and gB2 genotypes observed in our study group of fetuses were higher when compared to the rates of congenital infections in France or India [21, 26]. In turn, compared with data for congenital infections reported for American infants, the incidence of the gB1 genotype of HCMV was the same [19]. Based on our results and the literature data, the genotypic variability of HCMV seems to be geographically related. Besides single infections, several studies conducted for the United States, the Netherlands, Poland, and China showed the occurrence of congenital infections with multiple HCMV gB genotypes [14, 29–31]. The rates of mixed infections varied from approximately 2 % in Chinese to 45 % in American infants [31, 32]. In Poland, infections with multiple HCMV gB genotypes have been observed in 12.5 % of studied newborns [14]. In the current study population of fetuses and newborns, mixed infections were identified at the rate of 50.0 %, being similar to that for the American infants [32].

The mixed infections observed in our study fetuses were caused by the viral gB1–gB2 and gB2–gB3 genotypes, while they were caused by the gB1–gB2 genotypes in the newborns. A Dutch study performed in adults and infants reported the occurrence of double and triple mixed infections composed of various gB genotypes [29].

Among American infants, mixed infections were identified with viral gB1–gB2, gB2–gB3, as well as with gB2–gB4 genotypes [30]. Among infected Indian infants, the gB3 genotype was the most prevalent in symptomatic cases [26]. In turn, the gB2 genotype was more common in infants with long-term sequelae [26]. In Chinese infants, four gB genotypes were differentially distributed in patients with various symptoms of congenital cytomegaly [33]. Among congenitally infected Italian babies, HCMV disease and central nervous system (CNS) damage were more frequent among patients with gB1 and gB3 compared to those with gB2 and gB4 genotypes of HCMV; however, those differences were non-significant [18]. Taking into account the mixed infections, one study has shown no relationship between such infections and the clinical outcome of congenital cytomegaly [32]. Considering cytomegaly outcomes, we did not observe any differences among the fetuses and newborns infected with single and multiple HCMV genotypes. The small number of offspring with congenital cytomegaly and observed disease symptoms, as well as the uncertain significance of genotyping of a single HCMV glycoprotein B with extensive diversity among many other viral genes, seem to be the major causes of the observed lack of relationships. In this study population, we demonstrated a correlation between maternal HCMV genotypes and the viral genotypes identified in their fetuses and neonates. A similar relationship was also observed in Brazilian transmitting and non-transmitting mothers and their congenitally infected infants [34]. Similarly to other studies, we observed that the distribution of the gB genotypes in fetuses and neonates reflected the incidence of viral genotypes identified in the population of pregnant women [34]. Comparative to congenital infections, we also showed the gB2 and gB1–gB2 genotypes of HCMV present in the pregnant women to be the most common causes of single and mixed infections, respectively. No such study has yet been performed for any Polish population of pregnant women.

HCMV congenital cytomegaly diagnosed in Polish fetuses and newborns from Central Poland resulted from infections with gB2, gB1, and gB3 viral genotypes, with a distribution resembling the incidence rates of particular genotypes circulating in pregnant women. Similarly to other populations, both single and mixed infections may cause congenital cytomegaly, with asymptomatic as well as symptomatic outcomes.
